# Case report: A case of injury to the infrapatellar branch of the saphenous nerve caused by medial approach in knee arthroscopy

**DOI:** 10.3389/fneur.2023.1083871

**Published:** 2023-03-03

**Authors:** Jiyang Tan, Xunhao Wang, Fei Xiong, Jun Qian, Qiuwen Ying, Jingyi Mi

**Affiliations:** ^1^Department of Sports Medicine, Wuxi 9th People's Hospital Affiliated to Soochow University, Wuxi, Jiangsu, China; ^2^Medical College, Soochow University, Suzhou, China

**Keywords:** saphenous nerve, knee arthroscopy, medial approach, pain, numbness

## Abstract

We present the case of a 72-year-old man who was referred to our department for treatment of pain on the anteromedial infrapatellar side of the right knee with sensory disturbance that began 2 years earlier. The patient previously underwent right knee arthroscopy at another hospital for a meniscus injury 2 years earlier, which relieved his knee pain, but pain and discomfort near the incision of the medial portal persisted. Given this situation, various physical treatments, such as ice compress, were administered postoperatively. However, the symptom was only partially relieved before discharge. Subsequently, the patient visited two other hospitals and began taking oral pregabalin and duloxetine for treatment of the pain based on a diagnosis of right common peroneal nerve injury. The pain in the same dermatomal distribution was slightly relieved, but a withdrawal reaction was observed. However, the results of an ultrasound at our hospital indicated that the right medial quadriceps femoris tendon showed a hypoechoic area suggesting inflammatory changes. Physical examination of the right knee detected atrophy of the quadriceps femoris muscle, decreased muscle strength (M4), obvious tenderness in the medial side, radiating pain along the anterior tibia, and sensory disturbance (S3+); the results of a drawer test, McMurray test, pivot shift test, and lateral stress test were negative. Based on the aforementioned evidence, a diagnosis was made of injury to the infrapatellar branch of the saphenous nerve, after which neurolysis of the nerve in question was carried out. An enlarged incision was made along the original medial approach. Scar hyperplasia was observed after careful separation of the subcutaneous tissue. During neurolysis, branches were found wrapped in the scar; their continuity and integrity were confirmed after relief. The released nerve was placed in a physiological position. The patient's pain was clearly relieved, and numbness disappeared on the first postoperative day. At 1-month follow-up, all symptoms were found to have resolved.

## Introduction

Injury to the infrapatellar branch of the saphenous nerve (IBSN) can be triggered by many factors, including arthroscopic portals, incision for drilling of the tibial tunnel or dissection, and graft harvest. Most studies report that IBSN injury IBSN leads to sensory comorbidities in most cases. Problems can include loss of sensation, paresthesia, neuralgia, or hypersensitivity in the medial infrapatellar area of the lower extremity (1). Persistent pain after knee arthroscopy is rarely reported. We recently admitted a patient with pain on the anteromedial infrapatellar side of the right knee due to knee arthroscopy.

## Case presentation

### Chief complaints

A 72-year-old man presented with complaints of pain on the anteromedial infrapatellar side of the right knee with sensory disturbance that began 2 years earlier.

### History of present illness

The patient previously underwent right knee arthroscopy at another hospital for a meniscus injury in May 2020, which gave him relief from knee pain, but pain and discomfort near the incision of the medial portal persisted. Given this situation, various physical treatments, such as ice compress, were administered postoperatively. However, the symptom was only partially relieved before discharge. Subsequently, the patient visited two other hospitals and began taking oral pregabalin and duloxetine for treatment of the pain based on a diagnosis of right common peroneal nerve injury. Pain in the same dermatomal distribution was slightly relieved, but a withdrawal reaction was observed. The patient still felt migratory pain at the inner foretibia.

### History of past illness

The patient had high blood pressure for 5 years and had no other history of chronic disease or surgery.

### Family history

The patient's parents were deceased, cause of death unknown; he had one brother in good health. The patient denied (1) any similar disease in the family; (2) tuberculosis, hepatitis, or other infectious diseases in the family; and (3) hereditary or familial diseases, such as diabetes or hemophilia, in the family.

### Physical examination

Physical examination of the right knee detected atrophy of the quadriceps femoris muscle, decreased muscle strength (M4), obvious tenderness in the medial side, radiating pain along the anterior tibia, sensory disturbance (S3+), and positive Tinel's sign. The results of a drawer test, McMurray test, pivot shift test, and lateral stress test were negative.

### Imaging examinations

Color ultrasound and magnetic resonance imaging (MRI) examination of the patient's right knee were performed. Color ultrasound showed a hypoechoic area suggesting inflammatory changes in the right medial quadriceps femoris tendon. MRI showed level II injury in the posterior horn of the medial meniscus of the right knee joint and edema of the infrapatellar fat pad.

### Final diagnosis

The final diagnosis was injury to the infrapatellar branch of the saphenous nerve.

### Treatment

**Figure 1:**
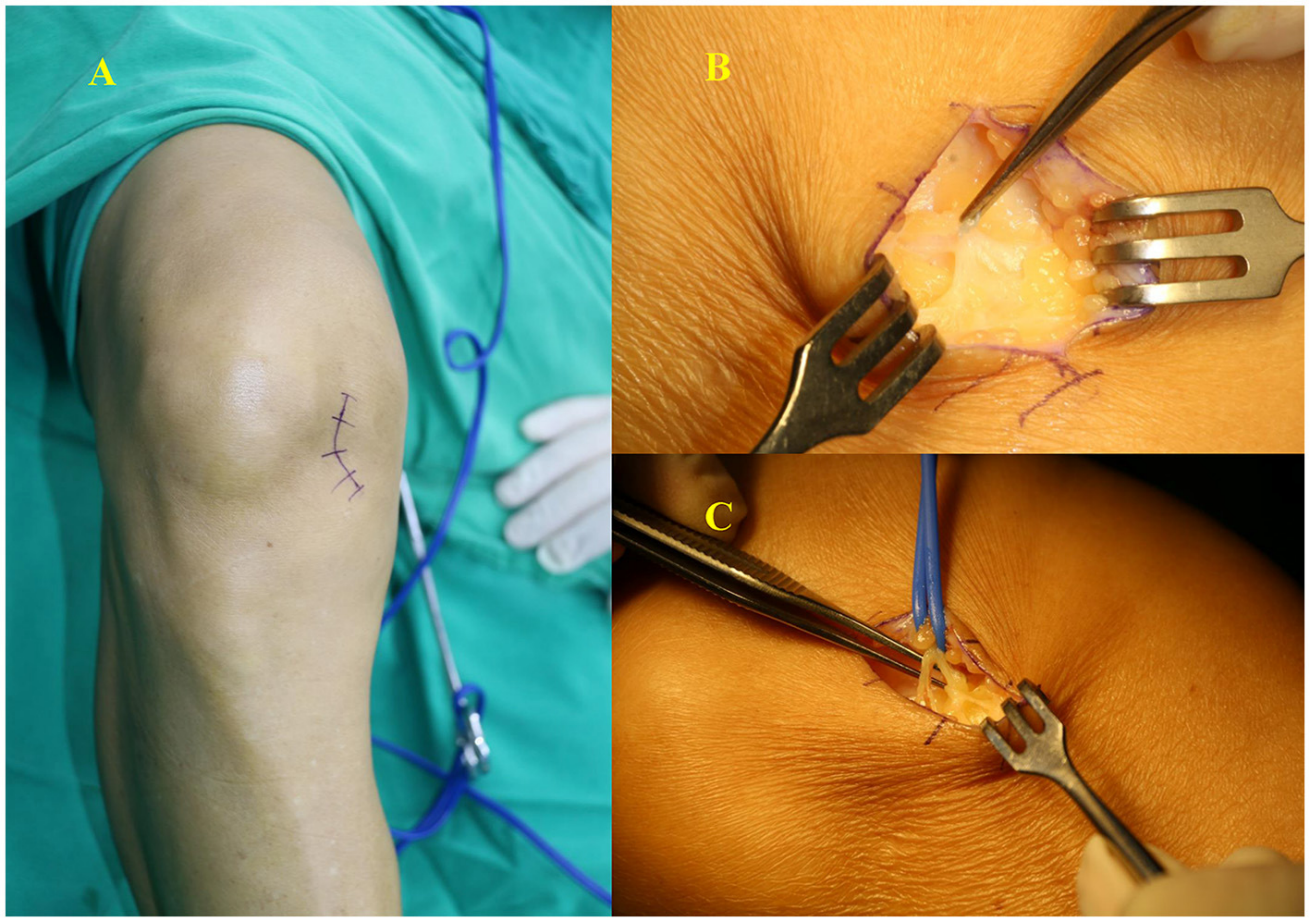
**(A)** An enlarged incision was made along the original medial approach. **(B)** Branches were found wrapped in the scar. **(C)** Continuity and integrity of the IBSN were confirmed after relief.

The patient underwent neurolysis for the infrapatellar branch of the saphenous nerve under epidural anesthesia in July 2022. An enlarged incision was made along the original medial approach. Scar hyperplasia was observed after careful separation of the subcutaneous tissue. During neurolysis, branches were found wrapped in the scar; their continuity and integrity were confirmed after relief. We translocated the nerve and embedded it under the fat (Figure 1). After surgery, the patient took 0.5 mg mecobalamin orally three times per day for 4 weeks.

### Outcome and follow-up

The patient's pain was clearly relieved, and numbness disappeared on the first postoperative day. One month after surgery, the pain had disappeared completely and the patient was satisfied with the outcome.

## Discussion

The saphenous nerve is the longest cutaneous branch of the femoral nerve. It divides into the IBSN, which serves the proximal tibia inferior medial to the patella (2, 3). The infrapatellar branch of the saphenous nerve splits off from the saphenous nerve in a highly variable way. It penetrates the sartorius muscle, after which it runs on a superficial course and generally forms two branches. Both branches cross the patellar tendon in the transverse direction to form the infrapatellar plexus. The infrapatellar branch of the saphenous nerve innervates the anteromedial aspect of the knee, the anterolateral aspect of the proximal part of the lower leg, and the anteroinferior part of the knee joint capsule (4–6). There are many reports of IBSN injury caused by surgery, especially knee arthroplasty (7), tendon extraction incision for anterior cruciate ligament reconstruction (8), and incisions for anteromedial knee surgery (9). Among these reports, the proportion of IBSN injuries caused by total knee replacement is 50–100% (10). Because the IBSN is highly variable, nerve damage in this area is considered inevitable (11). The most common symptoms associated with IBSN injury are hypoesthesia, numbness, or allergy in the anteromedial knee joint (12). Usually, these symptoms trouble the patient only for a short time, but in this case, the symptoms continued over a long period of time. We believe that the patient's IBSN, which was not completely disconnected, was the reason for the continued presence of sensory conduction; therefore, the patient's symptoms were primarily pain rather than numbness. The presence of scarring around the nerve, trapping the nerve, was what caused the symptoms to persist.

Prior to surgery, we had suspected subpatellar fat pad inflammation to be the cause of the patient's pain, but it was not until we observed positive Tinel's sign that the diagnosis was biased toward nerve damage or neuroma formation. However, this patient only underwent arthroscopic meniscus formation, and no large surgical incision was found; this made our diagnosis questionable, because the incision made by arthroscopic portal is small, and IBSN injury due to arthroscopic portal is rarely reported (13). Therefore, we used ultrasound to examine the affected area before the surgery (14). Unfortunately, ultrasound did not detect the formation of neuroma. Eventually, we had to make an enlarged incision along the original incision, and finally discovered the trapped nerve.

In general, symptoms of nerve entrapment do not progress rapidly, and there are few reports of neuroma formation leading to persistent pain 1 week after surgery. However, this patient felt pain 1 day after surgery, with a VAS score of up to 5. We do not believe that direct injury to the nerve was caused by the arthroscopic portal, but rather that the cause was unintended irritation of the nerve by the suture used to close the portal. Over the course of a few weeks, the sutures were absorbed and caused scarring around the nerve, which further perpetuated the symptoms. Therefore, we believe that the arthroscopic approach does not need to pursue aesthetics too strongly, and the epidermis can be directly sutured with less irritating sutures, without the need to apply too much suturing to subcutaneous tissue.

Krijgh et al. dissected 18 cadavers from adult donors and found a total of 23 infrapatellar branches. Based on this, they argued that the origin of the infrapatellar branch from the saphenous nerve is highly variable, as well as the position at which the infrapatellar branch passes the knee joint (15). This makes IBSN injury difficult to avoid. Gousopoulos et al. dissected the space between the subcutaneous and the capsule; this dissection is performed by grasping and knotting the sutures through the anteromedial portal to avoid iatrogenic saphenous nerve injury (16). Although there are many ways to avoid nerve injury, diagnosis and early treatment after injury are more important.

There are many ways to diagnose IBSN. Riegler et al. have reported that ultrasound successfully pinpoints the variable course of the IBSN from the origin to the most distal point and may therefore enable the correct identification of (iatrogenic) nerve damage in any location (17). We also initially used ultrasound to examine the patient's nerves after suspecting IBSN injury. Unfortunately, no positive results were observed in our examination.

After knee arthroplasty and other procedures that can lead to IBSN injury, denervation is usually the choice of most surgeons if IBSN injury occurs (18). The current conservative treatment for IBSN neuritis is traditionally performed with nerve blocks containing a steroid and a local anesthetic (19). Reports of cryoneurablation have also been published (20). Existing nonsurgical treatments can provide symptomatic relief but with variable long-term outcomes.

This case report still has several shortcomings. First, our follow-up time for this patient was short, and the long-term outcome remains unclear. Second, we have reported on only one case, so we are unable to present more useful information, such as Guidance on how to avoid IBSN injury and the incidence of IBSN injury.

## Conclusion

In conclusion, we should always be vigilant in relation to the possibility of IBSN injury during surgery, since there is a risk of saphenous nerve injury even if only a 1-cm long incision is made during arthroscopic surgery. The symptoms after an IBSN injury can easily confuse clinicians and make treatment difficult.

## Author's note

The authors have read the CARE Checklist (2016) and the manuscript was prepared and revised according to the CARE Checklist (2016).

## Data availability statement

The datasets presented in this article are not readily available because of ethical and privacy restrictions. Requests to access the datasets should be directed to the corresponding author.

## Ethics statement

The studies involving human participants were reviewed and approved by Wuxi 9th People's Hospital. The patients/participants provided their written informed consent to participate in this study. Written informed consent was obtained from the individual(s) for the publication of any potentially identifiable images or data included in this article.

## Author contributions

JT and XW reviewed the literature and contributed to drafting of the manuscript. FX and QY followed up the patients. JQ was the patient's first doctor. JM was responsible for revision of the manuscript for important intellectual content. All authors issued final approval for the version to be submitted.
